# Supplementary material for: Do you even exercise, ref? Exploring habits of Spanish basketball referees during practice and matches
Source: PeerJ. 2024 Jan 29;12:e16742. doi: 10.7717/peerj.16742 (PMC10832620; doi:10.7717/peerj.16742)
Supplement: Supplemental Information 3 [file peerj-12-16742-s003.pdf]

If so, could you indicate the reason(s) for physically reactivating during the break between halves?

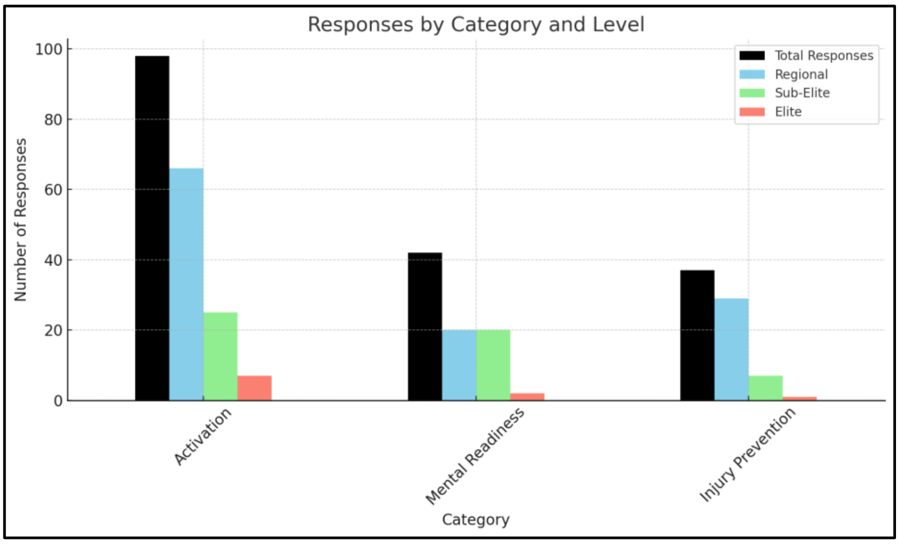

I. ACTIVATION

This category encapsulates referees' proactive engagement in re-warm-up activities during the break between halves to maintain or enhance their physical condition. Subcategories focus on different aspects of physical activation, from overall body readiness to specific actions preventing temperature loss in the body, muscles, and joints

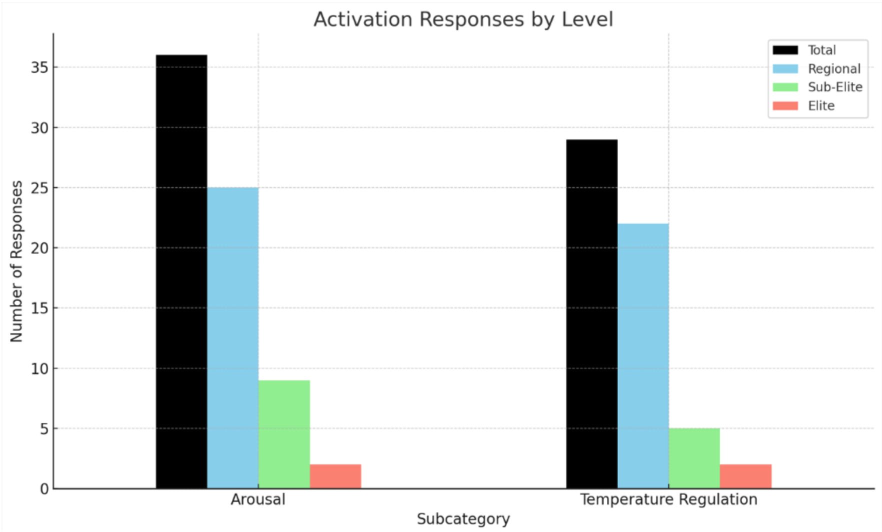

I.a. Arousal

This subcategory addresses referees' objectives in achieving overall bodily preparedness, ensuring optimal condition upon returning to the match.

REGIONAL

1. To avoid being inactive and return to the game in optimal condition.
2. To maintain my activity level.
3. Because I want to keep going.
4. To continue at the pace set before the break.
5. When tired from traveling, I need to warm up.
6. To reactivate my body and not be caught off-guard.
7. To maintain freshness and prevent the body from deactivating.
8. To re-prepare the body for physical exercise.
9. To enhance physical responsiveness.

10. To maintain the same muscle tone in the second half.
11. For muscle reactivation.
12. To avoid muscle atrophy.
13. To keep muscles and joints active.
14. Aim to reactivate ankles, back, legs…
15. To avoid relaxing the joints.
16. To increase heart rate before starting.
17. To stretch and avoid sitting.
18. To get back to 100%.
19. To be ready for the next quarter.
20. To be prepared post-break.
21. To be ready for the competition's restart.
22. For activation.
23. To stay active.
24. For maintenance and activation.
25. To maintain the level of activation reached during the game.

SUB-ELITE

26. To re-prepare the body for the activity.
27. To ready muscles for the second half's rhythm.
28. To try to maintain the same physical level and prevent temperature loss.
29. To re-enter the court with a high heart rate.
30. Staying active is crucial to avoid entering the second half too relaxed, which could lead to mistakes.
31. Teams take a break at crucial game moments, so staying activated is essential.
32. To stay active for post-rest start.
33. The need for reactivation.
34. To be active from the very first second.

ELITE

35. To activate my body quickly, yet gradually.
36. To enhance muscle tone.

I.b. Temperature Regulation

Focuses on referees' strategies to regulate body, muscle, and joint temperatures, aiming to prevent cooling, maintain optimal warmth, or increase temperature as needed. This ensures a seamless and effective transition into the match's second half.

REGIONAL

1. To avoid entering the game cold after the break. Just as players warm up during the break, referees must have their own routine.
2. To prevent temperature loss, I strive to stay active for the next half.
3. To avoid returning to the court with decreased temperature.
4. To avoid being cold, ensuring a smooth start to the second half.
5. To avoid running with decreased temperature.
6. To avoid starting cold in the second half.
7. To avoid starting off cold.
8. To avoid cooling down.
9. To counteract cooling.
10. To prevent cooling.
11. To continue staying warm.
12. To prevent the body from losing temperature, which can hinder starting the second half.
13. To restart the game at an optimal body temperature.
14. To prevent the body from cooling down.
15. To prevent muscle temperature loss and keep them tense and active.
16. To ensure muscles are ready to resume exercise without losing temperature.
17. To ensure muscles don't lose too much temperature.
18. Due to muscle cooling.
19. With a 10-minute break, I sometimes notice my knee and hip joints cooling. I feel more comfortable doing about a minute of dynamic stretching and joint mobility.
20. To prevent the body, especially the lower limb joints, from cooling.

21. To ensure joints don’t cool down and activity isn’t lost.
22. To prevent temperature loss in the joints.

SUB-ELITE

23. A 15-minute break is too long to stay warmed up without effort.
24. To avoid being cold after a significant 15-minute break.
25. To avoid entering the game cold.
26. To prevent the body from cooling
27. It's important to stretch and maintain warm muscles.

ELITE

28. To reactivate the body for the activity and avoid entering cold after a 15-minute break.
29. To avoid starting the third quarter cold and be prepared for physical activity, similar to the players.

II. MENTAL READINESS

This category reflects referees’ emphasis on psychological preparation during the break between halves, aiming for an appropriate level of concentration and focus. It underscores strategies to stay mentally agile and handle the cognitive demands of officiating, including enhancing decision-making and managing psychological stress, in the latter stages of the match.

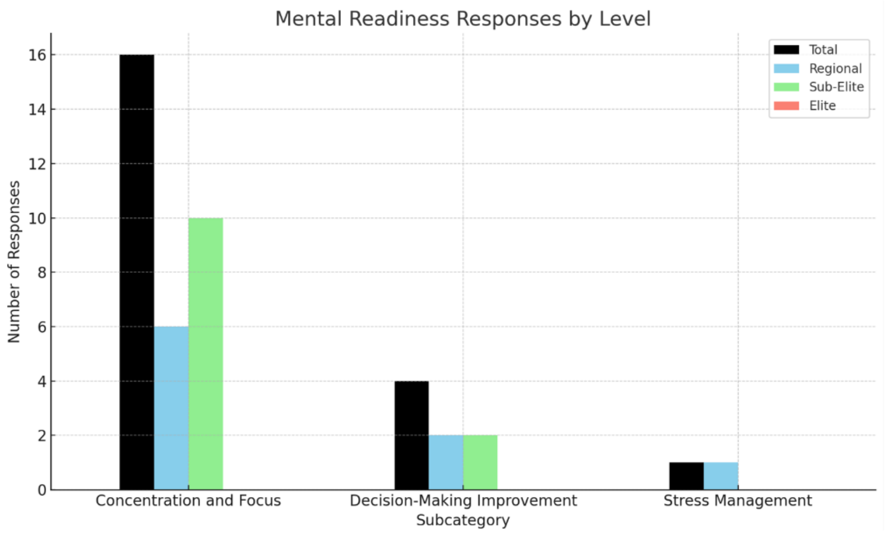

II.a. Concentration and Focus

Emphasizes the importance of achieving, maintaining, or regaining mental concentration and focus during the break between halves.

REGIONAL

1. For concentration.
2. For concentration.
3. For concentration.
4. To maintain connection with the game and stay on track.
5. To maintain focus.
6. To refocus on the game.

SUB-ELITE

7. A routine for concentration.
8. To start off concentrated.
9. For mental activation.
10. To enter focused in the third quarter.
11. The second part of the game is a mental challenge.
12. To maintain concentration in the initial minutes of the third quarter post-break.
13. To maintain the mental level achieved before the break.
14. The “reactivation” is crucial; there's still work to be done, and I can't afford to “disconnect” and start from zero.

15. To raise the concentration level again.
16. To refocus.

II.b. Decision-Making Improvement
Focuses on referees' efforts to maintain a state conducive to effective decision-making post-break.

REGIONAL

1. To remain unaffected and prevent impairments in my decision-making.
2. After the break is a critical moment to maintain control, like managing a scoring advantage, etc.

SUB-ELITE

3. To return to the game in an optimally activated state, thereby maintaining decision-making effectiveness in the third quarter.
4. To avoid returning to the court in a state unsuitable for properly assessing post-break actions.

II.c. Stress Management
This subcategory reflects the referees' efforts to regulate stress and pressure.

REGIONAL

1. To regulate stress and pressure.

III. INJURY PREVENTION
This category covers referees' strategies to prevent injuries during the match's second half, ranging from stretching to joint mobility exercises. It highlights their awareness of the physical demands of their role and their proactive steps to avoid injury and maintain well-being.

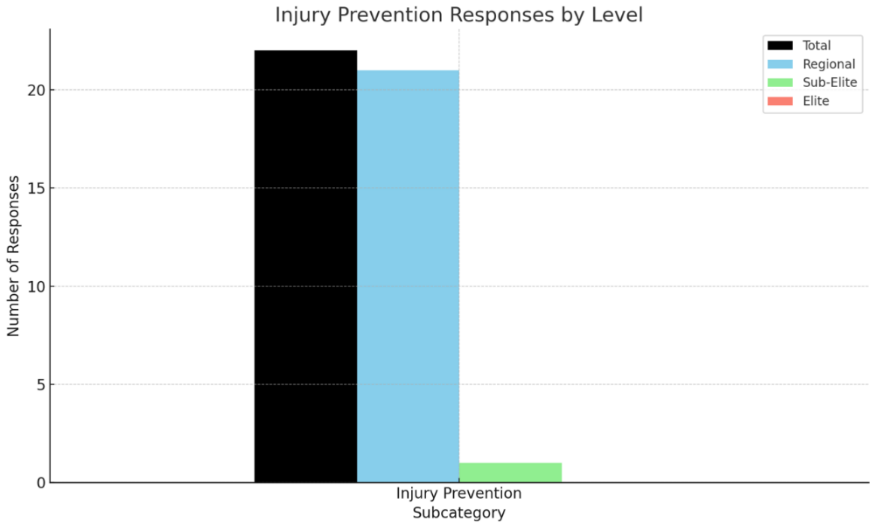

REGIONAL

1. To prevent injuries, muscle pulls…
2. To prevent muscle injuries.
3. I stretch to prevent muscle pulls.
4. Stretching limbs to prevent overloads.
5. Essential to stretch and warm up to prevent injuries.
6. To avoid getting injured during the game.
7. To avoid suffering injuries.
8. To avoid getting injured.
9. To prevent injuries.
10. To prevent injuries.
11. To avoid injuries.
12. To avoid injuries.
13. To avoid injuries.
14. To avoid injuries.
15. For well-being and to avoid injuries.

16. To prevent injuries and ensure a good recovery.
17. For physical recovery.
18. Due to fatigue.
19. To prevent discomfort in areas prone to stiffness or overload.
20. It's an issue that needs addressing, as improper restarts can lead to injuries.
21. If I don't stretch and work on joint mobility, I feel stiff and can't run as much or as comfortably.

SUB-ELITE

22. At 51 years old, I stretch between quarters and during breaks to prevent leg overload.

COMBINATION #1: ACTIVATION + MENTAL READINESS + INJURY PREVENTION

SUB-ELITE

1. To remain activated both mentally and physically for the second half of the game, thus preventing injuries and loss of concentration.

COMBINATION #2: ACTIVATION + MENTAL READINESS

REGIONAL

1. Habit of emotional release. Physical activity. Refocusing concentration.
2. Controlling my breathing aids mental relaxation and helps me reactivate both physically and mentally before returning to the court, essential for enduring the game's remainder and maintaining focus.
3. To re-enter with a good muscle temperature and maintain concentration from the start.
4. To enhance concentration and muscle activation.
5. For concentration and resuming muscle activation.
6. Two reasons: to enter both physically activated and focused
7. To avoid entering cold while refocusing.
8. To maintain both activation and concentration in the game.
9. To improve concentration and physically activate.
10. To stay focused and reactivate.
11. Physically activate to achieve the right level of concentration for the second half.

SUB-ELITE

12. Limited time, but after 8 minutes of inactivity, a few jumps or mobility exercises help me both physically and, especially, mentally activate.
13. To ensure body and mind stay active and focused during the break, avoiding disconnection and not starting the second half cold.
14. To be mentally and physically active after 15 minutes of rest.
15. To activate both mind and body for maximum performance.
16. To maintain concentration and physical intensity.
17. To enter the court actively and focused.

ELITE

18. For both physical and psychological reasons before resuming the game.
19. I get cold, and it helps me to refocus.

COMBINATION #3: ACTIVATION + INJURY PREVENTION

REGIONAL

1. To maintain muscle warmth and prevent injuries, while also activating the body for the game's continuation.
2. To avoid being cold and ensure joints are active, thus preventing injuries.
3. To avoid cooling down, thus preventing injuries.
4. To prevent returning post-break with lower temperature and potential injury.
5. To keep the body warm and prevent injuries.
6. To give 100% right after the break and avoid injuries.
7. To avoid injuries and maintain physical tension.
8. The body relaxes and because we will continue to make great physical effort, it is good to reactivate the body to avoid injury.

SUB-ELITE

9. The main reason is to return to the court at full capacity and prevent injuries.
10. To be physically ready in the game and prevent injuries.
11. To ensure muscles aren’t cold and to prevent injuries.
12. To reactivate muscles and prevent injuries.

ELITE

13. For muscle recovery and activation.

COMBINATION #4: MENTAL READINESS + INJURY PREVENTION

SUB-ELITE

1. To mitigate potential injuries and activate concentration.

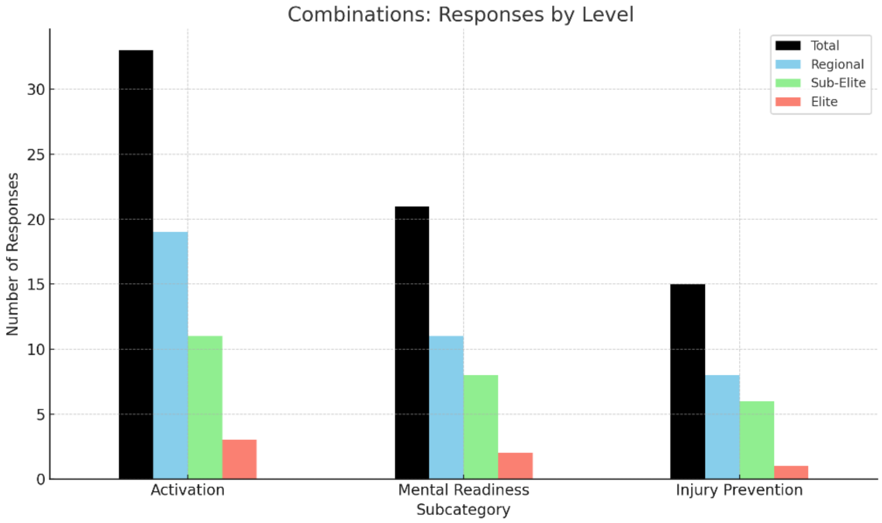

NOT VALID

This category could include responses that do not directly answer the question or are outside the scope of the study

REGIONAL

1. Mainly, I focus on mobility exercises and stretching.
2. To stretch legs and arms.
3. Engaging in jumps, running in the hallway, and active stretching.
4. Engaging in stretching.
5. To prevent significant changes.

SUB-ELITE

6. Engaging in stretching.

ELITE

7. Routine.

If not, could you indicate the reason(s) for not reactivating physically during the break between halves

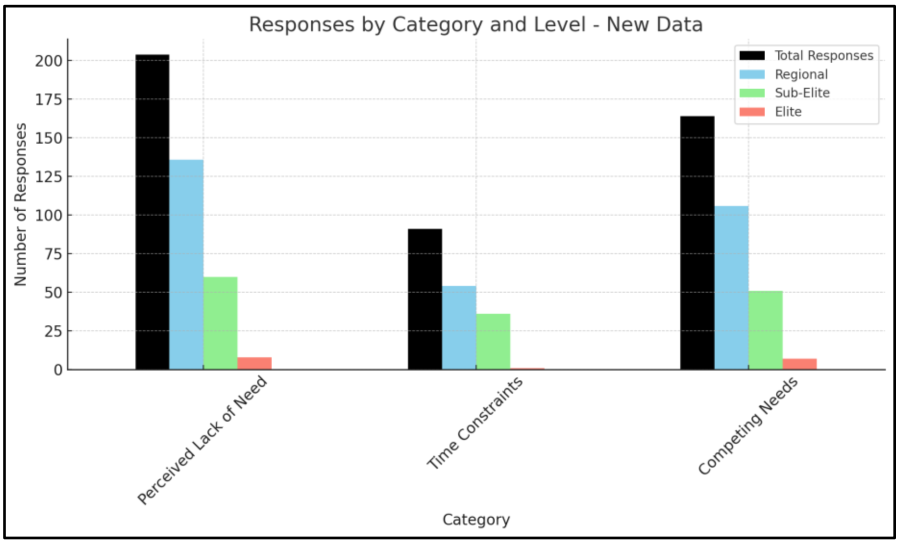

I. PERCEIVED LACK OF NEED

This category consolidates the stance among referees that additional physical reactivation during the break between halves is unnecessary. It includes sub-themes depicting a distinct mindset from enduring physical and mental preparedness or inherent self-sufficiency, to the absence of a routine or fundamental recognition of re-warm-up concept.

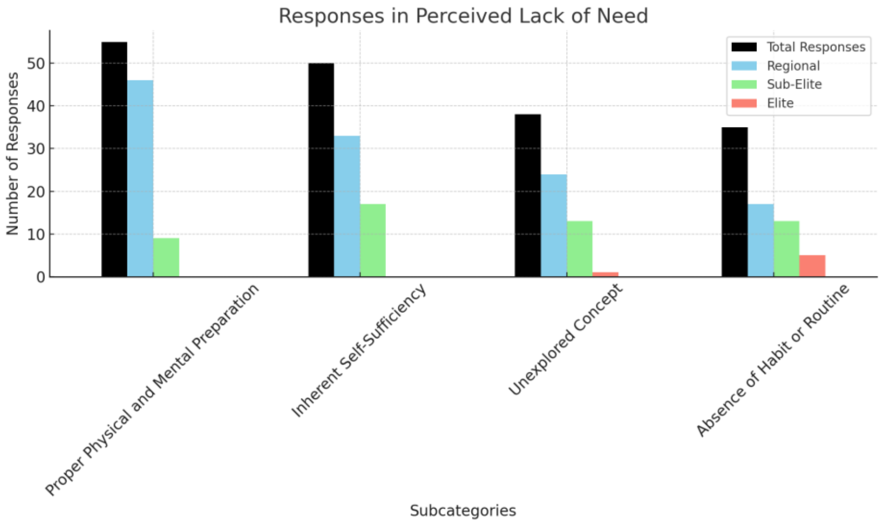

I.a. Proper Physical and Mental Preparation

Describes referees' belief in their ongoing physical and mental readiness during the break between halves, diminishing the necessity for additional physical reactivation.

REGIONAL

1. The breaks last at most 3 minutes, so I don't cool down much in this interval.
2. A 3-minute rest doesn't necessitate reactivation.
3. Because my breaks are only 3 minutes, as the highest level I've officiated involves children, it's not necessary for me.
4. I don't usually find it necessary, as most matches have short breaks of 3 minutes.
5. When the break is 10 minutes, I don't see it as strictly necessary to reactivate.
6. With just 10 minutes of rest, I don't see it as necessary.

7. The rest doesn't last longer than 10 minutes; there's no time to deactivate, though I do stretch.
8. It's not something I think about. The rest period is short, so I don't cool down.
9. There isn't enough time to lose my active state.
10. I understand that the rest period isn't long enough for the body to fully relax; I've never done it.
11. The rest period isn't long enough for me to cool down physically, so I don't need it.
12. Because in that time, I don't cool down significantly.
13. I always feel active; I typically don't lose my readiness during the break.
14. I believe I maintain my physical readiness throughout those 10 minutes.
15. I feel active from the first minute.
16. Even while resting, my body generally remains active.
17. I believe that I stay physically active.
18. I'm already in an active state.
19. I remain active.
20. I am already activated.
21. I stay active during the break, ensuring not to relax too much.
22. I don't need to do it; I usually stay active even while resting.
23. I'd never considered it. I tend to remain active in the third quarter without needing to exercise.
24. I believe I have sufficient readiness to start the second half without needing extra preparation.
25. I feel well-prepared to continue.
26. I feel good and don't think I need it.
27. Reactivation isn't necessary for me to be able to run again.
28. I don't experience a physical downturn that would require reactivation.
29. Because I don't completely cool down during the break, and I'll be warmed up again in a couple of plays.
30. I don't need it. My body doesn't cool down, and I quickly become active in the game's initial plays.
31. I typically don't cool down during breaks.
32. Because I don't cool down during the break.
33. I don't believe the body cools down enough to necessitate it.
34. Owing to my proper physical condition.
35. Because I'm physically prepared to respond if necessary.
36. My physical fitness keeps me prepared for the game.
37. Physically, I feel fine and don't see the need for reactivation, though I could be mistaken.
38. When physically fit for the game's demands, I haven't considered these exercises necessary.
39. I do stretching, but I don't necessarily follow a reactivation routine, as the kids I referee are not very physically developed
40. Probably, albeit unflattering, due to laziness. At 25, I don't feel the need to warm up again before running...
41. I've never thought physical reactivation necessary. I do mentally reactivate, but it's just a brief, one-minute exercise.
42. Because I quickly regain my focus in the first two plays of the quarter.
43. So far, I haven't needed it to maintain my concentration.
44. The game's intensity means you can't disconnect during the break, so there's no need to reconnect.
45. The pace of the matches in this category doesn't require as much.
46. I don't see it as necessary for the level of refereeing I do. However, I'm aware that a brief run (10 minutes) before the game is advisable.

SUB-ELITE

47. I remain active and don't struggle to continue the game at the same pace.
48. Physically, I'm active and quickly regain sensations in the first run.
49. I believe that at that point in the game, I am already physically activated.
50. I understand that my body remains active after 10 minutes of rest.
51. I don't think I lose activation during the break.
52. I don't believe I need physical reactivation.
53. If I don't feel physically burdened, I usually don't do it.
54. Because I don't consider it, and I'm physically well (both currently and in past seasons), I don't need reactivation. Just like players rest, I think it's appropriate for referees to do the same during those 10 minutes.
55. The physical exertion required for officiating doesn't seem excessive, so I usually don't warm up or reactivate during the break.

I.b. Inherent Self-Sufficiency
This subcategory reflects referees' belief in their inherent ability to maintain readiness without the need for additional physical reactivation.

REGIONAL

1. Believing I don't need physical reactivation, as I've never done it.
2. In my case, I don't consider it necessary to physically reactivate before resuming the game.
3. I typically don't need to reactivate.
4. Typically, I don't find it necessary.
5. I don't find it necessary, or haven't deemed it so.
6. I don't find it necessary.
7. I generally don't consider it necessary in most matches.
8. It's usually not necessary.
9. I hadn't deemed it necessary.
10. Because I don't need it.
11. I don't require it.
12. I don't require it.
13. It's not necessary.
14. I don't find it necessary.
15. I don't need it.
16. I don't believe it's necessary.
17. I don't consider it sufficiently necessary.
18. I don't view it as particularly necessary.
19. So far, I haven't felt the need.
20. The idea of not needing to warm up.
21. I didn't think it was necessary.
22. I don't see it as necessary.
23. I don't perceive a need for it.
24. Because I don't consider it necessary.
25. I don't need it.
26. I haven't needed it so far.
27. I sincerely believe I don't need it.
28. I believe I don't need reactivation.
29. I don't need it.
30. Currently, based on my sensations, I haven't needed it.
31. I have neither the habit nor the necessity for it.
32. Of little importance.
33. I can't find it important.

SUB-ELITE

34. I don't believe I need it.
35. So far, I haven't felt a need for it.
36. Currently, I don't see it as necessary.
37. It's usually not necessary.
38. I understand that I don't need it.
39. I don't need it.
40. There is no need.
41. I do not need it.
42. I haven't needed it.
43. I've never felt the need for it.
44. I don't see it as necessary.
45. I don't think it's necessary.
46. So far, I don't see it as necessary.
47. I haven't considered it necessary.
48. It doesn't seem necessary.
49. I haven't found it to be necessary.
50. I don't have that routine as I don't consider it necessary.

I.c. Unexplored Concept
Highlights referees' lack of previous consideration or familiarity with the concept of re-warm-up.

REGIONAL

1. I had never thought of doing it, though I now recognize it might be necessary.
2. I hadn't considered it before, but I might from now on.
3. I had never considered it necessary, nor had I thought about reactivation.
4. I've never needed it; in fact, I haven't even thought about it.
5. Exercising during rest hasn't been a consideration.
6. I haven't contemplated whether I need it.
7. I've never given it thought.
8. I I'd never considered engaging in any kind of activity during this game phase.
9. I have never thought about it, nor has anyone explained to me the need to do it.
10. I've never contemplated this option.
11. I've never given it thought.
12. I hadn't thought about it.
13. I hadn't thought about this.
14. I've never considered it.
15. I've never considered it.
16. I hadn't considered it.
17. I don't take it into consideration.
18. It's not something I've ever considered.
19. I have never thought about doing reactivation exercises.
20. Unaware of its necessity.
21. Lack of awareness.
22. Due to ignorance.
23. Unsure of the ideal warm-up for such situations.
24. I'm uncertain about what to do.

SUB-ELITE

25. I've never considered it, nor have I felt the need to do it.
26. I've never considered it.
27. I've never considered it, and thus never do it.
28. Honestly, I've never considered it.
29. I've never considered it.
30. I've never considered it.
31. I had never thought about it.
32. I had not taken it into account.
33. I've never seen a referee reactivate after a break, which has influenced my lack of consideration for it. Reading this, however, I now think it might be a good idea to ensure 100% readiness at the start of the 3rd quarter.
34. Missing a beneficial guide or routine for my activity.
35. Unaware of necessary routines.
36. Lack of knowledge.
37. Unawareness and lack of understanding.

ELITE

38. I'm unsure of the exercises needed for activation. I recognize the necessity but don't know the specifics.

I.d. Absence of Habit or Routine
This subcategory highlights the referees' lack of engagement in re-warm-up activities, attributed primarily to the absence of established habits or routines. It underscores their tendency to adhere to existing practices or deliberately avoid minimal-effort activities.

REGIONAL

1. It's more a matter of lack of habit than lack of time.
2. Lack of habit.
3. Lack of habit.

4. Habits.
5. I lack the routine for it.
6. Due to lack of habit, or never attempting it.
7. I haven't developed the habit.
8. Presumably, a lack of routine.
9. Absence of a routine.
10. It's not a habit for me.
11. Bad habit
12. Bad habit.
13. Laziness, as it involves activities that require minimal effort (jogging and mobility exercises).
14. Perhaps it's due to laziness.
15. Laziness.
16. Laziness.
17. Reluctance.

SUB-ELITE

18. I've never done it, but now that I'm considering the question, perhaps I should…
19. No habitual practice.
20. Lack of habit.
21. Lack of habit.
22. Lack of habit.
23. Lack of habit.
24. Absence of a routine.
25. Not a part of my routine.
26. Not part of my routine.
27. Not a routine.
28. Not habitually done.
29. Never practiced it as a routine.
30. I utilize the time for other purposes.

ELITE

31. Lack of habit.
32. Not a habit.
33. Absence of habit.
34. It’s quite uncommon.
35. Due to a bad habit.

II. TIME CONSTRAINTS

Addresses the challenge of limited break duration, typically 3 to 10 minutes, which referees find insufficient for re-warm-up activities.

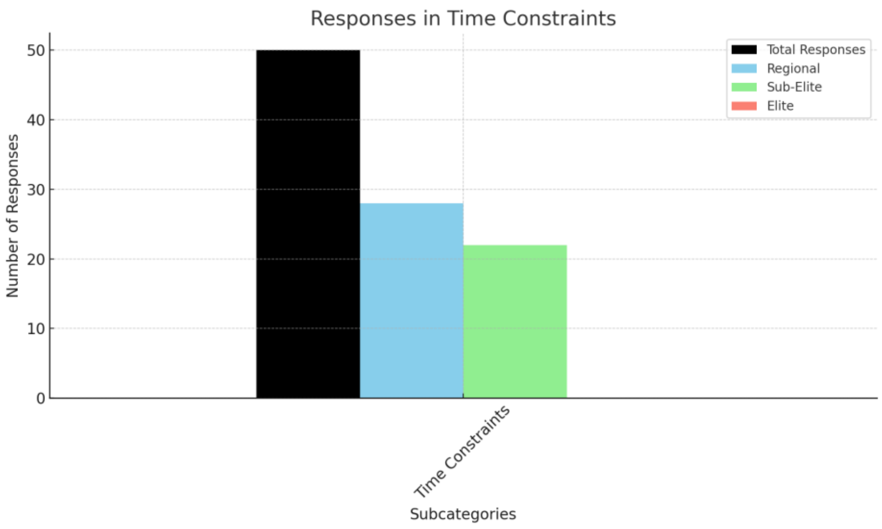

REGIONAL

1. Lack of time and space.
2. Currently, in the categories I officiate, there's only a 3-minute break between halves.
3. There's only a 3-minute rest period.
4. In just ten minutes, we don't have enough time.
5. Brief time window (10 minutes).
6. The time available is relatively brief (10 minutes).
7. The period is too short (less than 10 minutes).
8. The 10-minute break leaves no extra time.
9. Time constraints.
10. Time constraints.
11. Time constraints.
12. Insufficient time.
13. Insufficient time.
14. Lack of time.
15. Lack of time.
16. Lack of time.
17. Lack of time.
18. Lack of time.
19. Lack of time.
20. Lack of time.
21. Lack of time.
22. I don`t usually have much time.
23. Limited time during breaks.
24. Not enough time available.
25. Break time is limited.
26. There is not enough time for it.
27. The interval between halves is quite short.
28. I don't have a specific reason; due to time constraints, it's just not feasible for me.

SUB-ELITE

29. I should perform them to prevent cooling down after the break, but with only 10-minute breaks, there’s not enough time.
30. There’s not much time once you return to the locker room for a 10-minute rest.
31. The break is brief (5 minutes).
32. We lack time during the break.
33. Lack of time during the break.
34. Insufficient time to perform this reactivation.
35. Due to time constraints.
36. There is almost no time available.
37. I feel like there's no time for it.
38. Lack of time.
39. Lack of time.
40. Lack of time.
41. Lack of time.
42. Lack of time.
43. Lack of time.
44. No time available.
45. No time available.
46. Insufficient time.
47. Insufficient time.
48. Brief time.
49. Brief time.
50. Limited time.

III. COMPETING NEEDS

This category explores how referees balance their break time between technical-tactical preparation and personal rest and recovery needs.

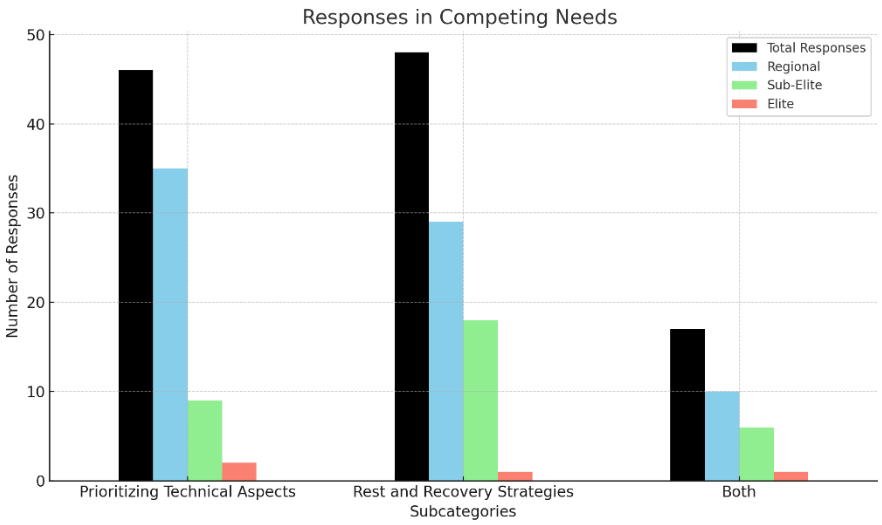

III.a. Prioritizing Technical Aspects of Refereeing

This subcategory underlines referees' prioritization of technical-tactical discussions during breaks, including game record reviews and strategic collaboration with colleagues.

REGIONAL

1. It would be beneficial if I did it, but in federated games, I mostly spend that time discussing the game and improvement strategies with my partner.
2. Game reporting, discussing the game, etc.
3. I prefer discussing game aspects with my colleagues.
4. I review the game's record up to that point and discuss the first half's incidents with colleagues.
5. Priority given to using that time for addressing technical refereeing issues.
6. Prioritize other issues related to the game.
7. Coordinating with the team.
8. I typically spend most of the time assessing the work done.
9. We usually use the rest period to talk with colleagues.
10. I typically use the break to review the match record, like fouls for each team, players with more fouls, etc.
11. Due to discussing the game with my partner.
12. I use it to discuss the game's progress with my partner.
13. I use the time for other tasks like reviewing the record, talking to my partner, etc.
14. I focus on discussing the game with my partner.
15. I prefer discussing the game with my partner.
16. I typically use the break to discuss various aspects of the game with my partner.
17. I attempt to communicate with my partner about the game's progress and aspects for my improvement.
18. To discuss the game with my partners.
19. Most of the break time is spent discussing with my partner how we are managing the game and what to focus on in the second half.
20. Because I use it to talk about the game situation.
21. Chatting.
22. I talk with colleagues.
23. Engaging in work-related activities.
24. Discuss with colleagues how we are handling the game.
25. We discuss with our partner how the game is progressing.
26. Discuss the game with my partner during the journey from the court to the locker room and back.
27. I speak about the development of the first quarter
28. I usually talk about technical aspects with the partner and/or check that everything is correct.
29. There is a 10-minute break, where the match report is reviewed, and aspects to be improved in the second half are discussed with the partner.

30. I usually dedicate it to discussing game aspects with fellow referees.
31. I seize the opportunity to discuss game improvement strategies with my partner.
32. I review the scoresheet and discuss the game with my partner.
33. Hydrate and discuss the game.
34. Review the scoresheet and chat with partner.
35. Chat among the referees.

SUB-ELITE

36. I use the break for game analysis and corrections.
37. I mentally reactivate with my partner by discussing the game and its progression.
38. I use the breaks for the match's technical work.
39. Discuss technical aspects of the match.
40. I typically use it to discuss improvements or chat with colleagues.
41. Preference for engaging in technical discussions with the partner.
42. I use the break to discuss game situations.
43. I focus on talking with my partner.
44. The reason is to analyze the previous two quarters.

ELITE

45. Engaging in discussions about the game with colleagues.
46. We focus on the game and its events.

III. b. Rest and Recovery Strategies

This subcategory emphasizes the referees' prioritization of restorative practices like stretching, hydration, nutrition, and mental relaxation during the break between halves. It reflects their understanding of the importance of conserving energy, sustaining mental focus, and achieving physical and mental relaxation to ensure peak performance in the match's latter stages.

REGIONAL

1. Limited places are available for this. With multiple games, conserving energy is preferable.
2. I aim to remain as non-fatigued as possible for subsequent games. If I feel a bit inactive, I do some mobility exercises.
3. I engage in stretching, but don't regard it as reactivation.
4. I do some stretching.
5. It's not routine; I just do some warm-up movements.
6. I remain standing or moving to prevent cooling down.
7. I mentally activate by visualizing the first half.
8. I prefer to maintain concentration in the game.
9. Focusing on concentration.
10. Focusing on concentration and mental relaxation.
11. Rest, followed by mental activation, especially with 3 minutes remaining before restart.
12. Reactivating between halves makes me tired and nervous, so I use breaks to relax.
13. I take time for mental relaxation, disconnecting from the game's progress to return with more focus.
14. I use the time for rest and mental relaxation.
15. My preference is for mental rest.
16. Rest and return to a calm state.
17. Rest and reflect.
18. The necessity to disengage both physically and mentally from the game.
19. Physical rest.
20. For muscle recovery.
21. I utilize that time for muscle relaxation.
22. I invest the time in recovery
23. Rest.
24. To extend my recovery period.
25. I dedicate the time to recovery.
26. Resting suits me better.
27. I avoid other activities since the body requires its rest periods.
28. My preference is to rest.
29. I recharge energy by eating a banana.

SUB-ELITE

30. Heel calcifications.
31. I prefer to recover and then gradually reactivate with some mobility once back on the court.
32. At most, I stretch a little and pat my legs to warm them, but it’s not the same as the pre-match warm-up.
33. To prevent cooling down, I strive to stay active for the second half.
34. My preference is for mental reactivation to maintain concentration, relegating the physical aspect.
35. I need 5 minutes to concentrate on the actions on the court (my physical preparation is a consistent, weekly effort).
36. Aim to prevent excessive fatigue.
37. Even though I know it’s useful, I usually use the time more for rest.
38. Time is solely allocated for rest.
39. I need to rest.
40. I prefer using it for recovery.
41. Because my preference is to rest.
42. I try to rest.
43. Start the second half of the game recovered.
44. I use it to lower my heart rate and rest.
45. Rest and muscle relaxation.
46. Body rest and hydration.
47. I typically visit the locker room between games to use the restroom or drink water.

ELITE

48. Habitually, I don’t do it. I focus more on resting than activation.

III. a+b. Prioritizing Technical Aspects of Refereeing and Rest and Recovery Strategies

Demonstrates how referees manage their time to address both game-focused discussions and personal recovery, ensuring physical and mental readiness for subsequent match phases.

REGIONAL

1. Rest while reviewing the game with my partner
2. Analyzing the match and physically relaxing.
3. Resting and discussing the game with my partner.
4. Resting and ensuring everything is in order.
5. Prefer to rest and discuss issues related to the game.
6. Resting, and often focusing on critical game aspects and problematic situations...
7. Comfort and the need to discuss game aspects.
8. I comment on the game with my partner, hydrate myself, and check how the record and other ongoing games are progressing.
9. I prefer discussing the first half with my partner, taking the opportunity to eat some fruit or bars, resting for a few minutes, and maintaining focus on the game.
10. It varies with each game's level and demand, and the physical load from the weekend. In 10 minutes, I usually opt for a break to review the game situation and plan for the second half. If I feel any physical discomfort, I try to stretch and warm up a bit.

SUB-ELITE

11. I typically use the break for teamwork and focus more on mental than physical aspects.
12. I use this time for mental rest and sharing impressions with colleagues rather than physical activation, except for a brief sprint to my position at the start of the third quarter.
13. I use it for rest, changing shirts, and exchanging feedback with colleagues.
14. I use the time to rest and talk with my partner.
15. My preference is to rest and chat with my partner.
16. I prefer eating fruit and discussing the game.

ELITE

17. I prefer a "cool down" and using the 5-7 minutes in the locker room to share thoughts with my partners.

COMBINATION #1: PERCEIVED LACK OF NEED + TIME CONSTRAINTS + COMPETING NEEDS

REGIONAL

1. In children's competitions, breaks are only 3 minutes, causing minimal interruption. In higher levels, when we're two referees, we spend the break reviewing the scoresheet and discussing the second half's strategy, leaving little time for extensive warm-ups. However, if time allows, I do try to actively stretch for at least a minute.
2. Rest, little time between halves, I have not lost temperature.

COMBINATION #2: PERCEIVED LACK OF NEED + TIME CONSTRAINTS

REGIONAL

1. There's a lack of time between halves and I don't consider reactivation necessary, as I stay active.
2. The rest period is brief, but I feel sufficiently prepared to start the second half.
3. Lack of time, laziness, etc.
4. Sometimes there's a lack of routine or time.

SUB-ELITE

5. The break is 10 minutes. With locker room visits and returning, 4-5 minutes pass, and we must be on court 3 minutes before starting, leaving only a few minutes in which I typically don't get cold.
6. I answered NO because I lack a routine. I do some stretching and brief activation, especially during long breaks. Normally, I don't lose warmth.
7. Due to lack of time and habit.
8. Due to lack of time and habit.
9. Habit and timing make consistent application challenging. Sometimes I reactivate, but often there's too little time, so it's not routine.
10. Laziness and lack of time.

COMBINATION #3: PERCEIVED LACK OF NEED + COMPETING NEEDS

REGIONAL

1. It's not necessary; I prefer to rest considering the numerous matches I officiate.
2. I never thought it was essential; I've always viewed the break as a time for rest.
3. Because I hadn't thought of it in that way, due to ignorance or rest.
4. I feel already immersed in the game's rhythm and use the break for rest.
5. I didn't see it as necessary since I receive feedback about the first half from my colleagues.
6. We usually go to the locker rooms to discuss technical issues, and within 10 minutes, I don't feel the need to reactivate.
7. I'm still somewhat warm, and my colleagues discuss game details.
8. Discussing refereeing and the game with colleagues, I don't regard it as very important.
9. Since I haven't formed a habit of reactivating after halftime, I usually use that time to discuss game issues with colleagues, use the restroom, and check the record.
10. Out of unawareness, I use the time to discuss game situations with my partner.

SUB-ELITE

11. I don't need it. I focus on breathing, hydrating, or eating a banana. In the locker room, my preference is stretching and resting while seated.
12. I use breaks for resting without considering reactivation.

ELITE

13. I don't see it as necessary; I prefer discussing the game with my partners.
14. I don't need it; I mentally prepare instead.

COMBINATION #4: TIME CONSTRAINTS + COMPETING NEEDS

REGIONAL

1. The break is too short to do these exercises after discussing technical aspects with the officiating team.

2. I often forget and run out of time due to discussing the game with partners, reviewing records, making corrections, preparing reports, planning for the next half, and bathroom breaks. All these tasks are time-consuming.
3. There's no specific reason; perhaps I can't find the time between visiting locker rooms, reviewing records, and so on.
4. Insufficient time for rest and coordination with the scoring table.
5. With only 10 minutes between quarters, spending time reviewing the record and discussing game aspects with my partner leads me not to consider reactivation.
6. There's no time; I use that moment to discuss game aspects with the referee team.
7. I find there's little time, which I use to talk with my colleagues.
8. The 10-minute break is used to discuss the game, hydrate, and review the record.
9. Given the short breaks, it's preferable to discuss the match's technical aspects with partners.
10. In provincial competitions, there's barely enough time to have a beverage, wipe off sweat, and ensure the points and record are correct.
11. The break is nominally 10 minutes, but effectively 7, as we return to the court 3 minutes before the second half. During these 7 minutes, we head to the locker room with our partner to discuss the game and recover (drinking water, eating something, etc.)
12. The break is short, and we use it to discuss the game's progress and prepare for the second half.
13. There's no time, as the game needs to be discussed and the record reviewed.
14. There's no time available. We dedicate it to discussing the match.
15. During the halftime break, when with a partner, we go to the locker room to review game situations, correct discrepancies, and discuss team strategies. After this, there's little time left due to other required duties.
16. Due to rest and limited time.
17. Lack of time between halves; we rest and drink water before the next half.
18. Due to fatigue, I prefer resting in lower categories with minimal time between halves.
19. Lack of time and fatigue.
20. Due to physical exhaustion and lack of time.

SUB-ELITE

21. With only 10 minutes of rest, talking with a partner and official discussions leave no time. The priority is game control.
22. We typically discuss the game, and 10 minutes of rest isn’t sufficient.
23. It's a matter of time. With only 10 minutes for the locker room visit, record review, and partner discussion, it’s complicated. Fifteen-minute breaks in other categories seem more feasible.
24. Lack of time due to reviewing the record, visiting locker rooms, and discussing situations with others.
25. Limited time available for reviewing the record and discussing with a partner.
26. Not much time available for reviewing the record, personal hygiene, and discussing the game's progress.
27. Limited time, which I prefer to spend chatting with my partner.
28. Going to the locker rooms and discussing the game with a partner only leaves time for a couple of sprints.

ELITE

29. No specific moment for activation. Typically, within the 10-minute locker room break, we discuss the game and then head to the court with 5 minutes left, doing nothing specific, though I advocate reactivation for greater assurance.

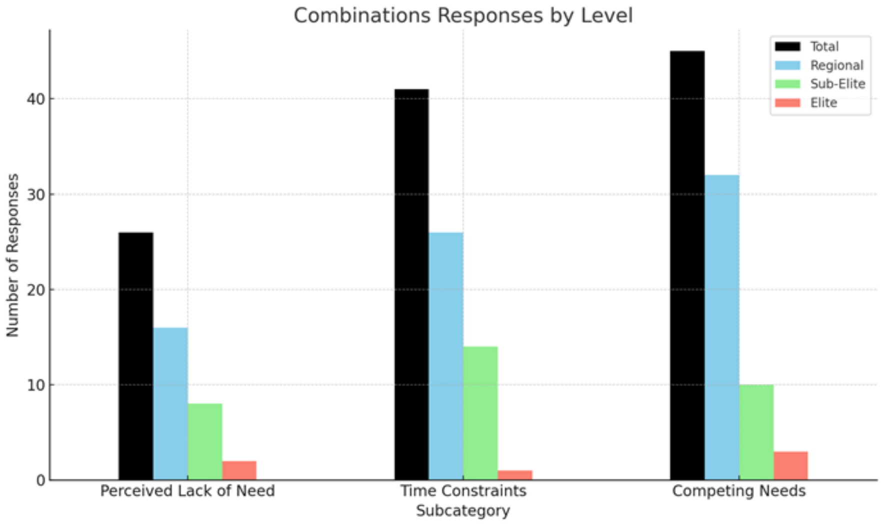

NOT VALID

This category could include responses that do not directly answer the question or are outside the scope of the study

REGIONAL

1. I don't think the break is necessary.
2. Remaining in the locker room during the break.
3. No reason, though I should do it.
4. Actually, I have no specific reason.
5. No particular reason.
6. No specific reason.
7. I'm a table officer.

SUB-ELITE

8. I have no specific reason, though I acknowledge I should do it.
9. None.
10. Activation is necessary upon entering the sports center.

ELITE

11. I do it, albeit without a routine.
