# Supplementary material for: Do you even exercise, ref? Exploring habits of Spanish basketball referees during practice and matches
Source: PeerJ. 2024 Jan 29;12:e16742. doi: 10.7717/peerj.16742 (PMC10832620; doi:10.7717/peerj.16742)
Supplement: Supplemental Information 5 [file peerj-12-16742-s005.docx]

**Physical exercise habits in basketball referees**

Dear colleagues,

We are conducting a study with the purpose of determining the nature of the physical exercise habits shown by Spanish basketball referees.

We are therefore seeking referees from this discipline who can contribute to the present study based on their experience.

It will only take 5 minutes to complete the questionnaire.

The collected data could be used to develop scientific reports and publications. All gathered data will remain confidential. The main researcher in charge of the study guarantees anonymity. As a participant in this study, you will have access to the findings of the research if so you wish.

We also ask you to help us distribute this questionnaire to the referees you know.

In order to obtain a broad picture of this issue, we would like to obtain as many responses as possible. Your collaboration is very important to us to continue progressing in our research and disseminating possible strategies.

Thank you very much for your collaboration.

**Declaration of informed consent.**

Having been informed of the aim and purpose of the research and the conditions of your participation, do you wish to participate?

- Yes
- No

**Personal and refereeing data**

Date of birth*

*DD****/****MM****/****YYYY*

Sex*

- Men
- Women

Height (cm)*

*Your answer*

Weight (Kg)*

*Your answer*

Place of residence (City, Province) *

*Your answer*

Referee experience*. You can indicate several options

- Local
- Regional
- National
- International

Please specify how many years of experience for each of the previously chosen options. For example: Local (20 years), Regional (19 years), National (10 years), International (no experience)*

*Your answer*

**Below, given the exceptional nature of the seasons affected by the COVID-19 situation, answer by thinking carefully about a "normal" season, typical pre-pandemic (for example, the 2018-2019 season) whenever possible.**

Based on what season are you going to answer the following questions? (whenever possible, answer based on a pre-pandemic season)*

- 2020-2021
- 2019-2020
- 2018-2019
- Other:

Type of refereed competitions*

- Senior category
- Youth categories

Weekly frequency of refereed games*

- 1 game/week
- Between 1 and 2 games/week
- Between 2 and 3 games/week
- Between 3 and 4 games/week
- Between 4 and 5 games/week
- Between 5 and 6 games week
- Between 6 and 7 games week
- 7 or more games/week

In which category have you officiated? (Spanish League, Group 1, Group 2, etc.). Indicate all the categories where you have refereed during the season you have previously chosen (for example, 2019-2020)

*Your answer*

**Physical exercise habits**

Do you engage in physical exercise (planned, structured, repetitive physical activity that promotes the maintenance or development of physical fitness) regularly? For example, running or ride a bike, working out at the gym, swimming, etc.? *

- Yes
- No

**If you answered NO, go to the activity questions in the competition (next section, 6 of 6). If you answered YES, please proceed with the following questions**

Do you have a personal fitness trainer?

- Yes
- No

What kind of physical exercise do you usually do? You can select more than one type:

- To maintain or develop aerobic endurance (swimming, cycling, running, jumping rope, etc.)
- To maintain or develop muscle strength (different muscle strengthening techniques with machines, with free weights, with your own body weight, with elastic bands, etc.)
- To maintain or develop speed (ability to perform sprints in the shortest possible time and to repeat them the greatest number of times)
- To maintain or develop flexibility (either with your own body, the help of another person, or with elements such as towels, elastic bands, foam rollers, etc.)
- To maintain or develop other abilities (coordination, balance, agility)

On a typical day, could you indicate how much time you spend on this physical exercise?

- Less than 15 minutes
- Between 15 and 30 minutes
- Between 30 and 45 minutes
- Between 45 and 60 minutes
- More than 60 minutes

In a typical week, could you indicate how many days a week you do this physical exercise?

- 1 day
- 2-3 days
- 4-5 days
- 6-7 days

**Activities during basketball competition**

Do you have any physical warm-up routine before starting to officiate a game? *

- Yes
- No

If so, how much time do you spend on this physical routine? Please indicate a specific number of minutes (for example, 3 minutes, or 20 minutes), or a range of minutes (for example, 5-10 minutes, or more than 15 minutes, etc.)

*Your answer*

During the break of the games (between halves), do you have an exercise routine to reactivate physically? *

- Yes
- No

If so, please indicate what kind of activities you perform (you can check several options)

- Jog or run.
- Running at maximum speed (sprinting).
- Strength exercise (jumps, push-ups, etc.).
- Stretches and joint mobility.
- Coordination and balance exercises.
- Breath management techniques (activation/relaxation, awareness).
- Other.

If so, could you indicate the reason(s) for physically reactivating during the break between halves?

*Your answer*

If not, could you indicate the reason(s) for not reactivating physically during the break between halves?

*Your answer*

In relation to stretching and/or joint mobility exercises, when do you perform them? (you can check several options) *

- Never
- During the initial warm-up
- At the break between the first and second quarters
- During the break between halves
- At the break between the third and last quarter
- At the end of the game

Finally, if you have answered that you never perform stretching and/or joint mobility exercises, could you indicate the reason(s) for this?

*Your answer*

**Acknowledgement**

As we mentioned previously, we appreciate the dissemination of this form among the referees you know in order to reach the largest possible number of them.

We provide the section below for those who want to make any comments or have any doubts or suggestions.

Thank you so much!

Contact: [dgonzd02@estudiantes.unileon.es](mailto:dgonzd02@estudiantes.unileon.es)

**Comments**

*Your answer*
