# Supplementary material for: Do you even exercise, ref? Exploring habits of Spanish basketball referees during practice and matches
Source: PeerJ. 2024 Jan 29;12:e16742. doi: 10.7717/peerj.16742 (PMC10832620; doi:10.7717/peerj.16742)
Supplement: Supplemental Information 6 [file peerj-12-16742-s006.docx]

**Hábitos de ejercicio físico en árbitros de baloncesto**

Buenos días a tod@s compañer@s!

Nuestro objetivo es conocer la realidad de los hábitos de ejercicio físico, realizado por los árbitros de baloncesto español.

Por ello, buscamos a árbitros de esta disciplina que puedan aportar su granito de arena.

Solo les llevará 5 minutos.

Los datos obtenidos tendrán carácter confidencial, asegurándose el anonimato en las respuestas y estarán a cargo del investigador principal para el posterior desarrollo de informes, así como para divulgación científica en revistas y publicaciones.

Al final del estudio, como participante podrá conocer los resultados generales del estudio, pues se harán públicos a través de informes y publicaciones en revistas científicas.

También les pediremos que nos ayuden a distribuir este cuestionario entre los árbitros que conozcan.

Nuestro objetivo es conseguir obtener el mayor número posible de respuestas para poder tener una visión lo más amplia posible sobre este tema. Para nosotros es muy importante vuestra colaboración para continuar avanzando en la investigación y divulgación de posibles estrategias.

Muchísimas gracias por su colaboración.

**Declaración de consentimiento informado.**

Habiendo sido informado/a del objetivo y finalidad de la investigación, y de las condiciones de su participación, ¿deseas participar?

- Sí
- No

**Datos personales y de arbitraje**

Fecha de nacimiento *

*Mes/día/año*

Sexo*

- Hombre
- Mujer

Altura (cm)*

*Tu respuesta*

Peso (Kg)*

*Tu respuesta*

Lugar de residencia habitual (localidad, provincia) *

*Tu respuesta*

Experiencia de arbitraje *. Puede indicar varias opciones

- Local
- Regional
- National
- International

Por favor, precise cuántos años de experiencia para cada una de las opciones anteriormente elegidas. Por ejemplo: local (20 años), regional/autonómica (19 años), nacional (10 años), internacional (sin experiencia) *

*Tu respuesta*

**A continuación, dada la excepcionalidad de las temporadas afectadas por la situación de COVID-19, conteste pensando con detenimiento en una temporada "normal", típica pre-pandemia (por ejemplo, temporada 2018-2019) siempre que sea posible.**

¿En base a que temporada va a responder las siguientes preguntas? (siempre que sea posible, conteste basándose es una temporada pre-pandemia) *

- 2020-2021
- 2019-2020
- 2018-2019
- Otro:

Tipo de competiciones arbitradas *

- Categoría senior
- Categorías inferiores

Frecuencia semanal de partidos arbitrados *

- 1 partido/semana
- Entre 1 y 2 partidos/semana
- Entre 2 y 3 partidos/semana
- Entre 3 y 4 partidos/semana
- Entre 4 y 5 partidos/semana
- Entre 5 y 6 partidos semana
- Entre 6 y 7 partidos semana
- 7 o más partidos/semana

¿En qué categoría ha arbitrado? (FEB, grupo 1, grupo 2, etc.). Indique todas las categorías donde ha arbitrado durante la temporada que ha elegido previamente (por ejemplo, 2019-2020)

*Tu respuesta*

**Hábitos de ejercicio físico**

¿Realiza usted ejercicio físico (actividad física planificada, estructurada, repetitiva y que favorece el mantenimiento o el desarrollo de la aptitud física) habitualmente? Por ejemplo, salir a correr o en bicicleta, entrenar en el gimnasio, nadar en la piscina, etc.? *

- Sí
- No

**Si ha contestado NO, pase a las preguntas de actividades en la competición (siguiente sección, 6 de 6). Si ha contestado SÍ, por favor, proceda con las siguientes preguntas**

¿Tiene usted preparador físico personal?

- Sí
- No

¿Qué tipo de ejercicio físico realiza habitualmente? Puede seleccionar más de un tipo

- Para mantener o desarrollar la resistencia aeróbica (natación, ciclismo, carrera, saltar a la comba, etc.)
- Para mantener o desarrollar la fuerza muscular (diferentes técnicas de fortalecimiento muscular con máquinas, con peso libre, con el propio peso corporal, con gomas elásticas, etc.)
- Para mantener o desarrollar la velocidad (capacidad de realizar sprints en el menor tiempo posible y de repetirlos el mayor número de veces)
- Para mantener o desarrollar la flexibilidad (ya sea con el propio cuerpo, ayuda de otra persona, o con elementos como toallas, gomas elásticas, foam roller, etc.)
- Para mantener o desarrollar otras capacidades (coordinación, equilibrio, agilidad)

En un día típico, ¿podría indicar cuánto tiempo dedica a este ejercicio físico?

- Menos de 15 minutos
- Entre 15 y 30 minutos
- Entre 30 y 45 minutos
- Entre 45 y 60 minutos
- Más de 60 minutos

En una semana típica, ¿podría indicar cuántos días a la semana realiza este ejercicio físico?

- 1 día
- 2-3 días
- 4-5 días
- 6-7 días

**Actividades durante la competición de baloncesto**

¿Tiene usted alguna rutina física de calentamiento antes de comenzar a arbitrar un partido? *

- Sí
- No

¿En caso afirmativo, cuánto tiempo dedica a esta rutina física? Por favor, indique una cantidad de minutos concreta (por ejemplo, 3 minutos, o 20 minutos), o un rango de minutos (por ejemplo, 5-10 minutos, o más de 15 minutos, etc.)

*Tu respuesta*

¿Durante el descanso de los partidos (entre mitades), tiene usted una rutina de ejercicio para reactivarse físicamente? *

- Sí
- No

En caso afirmativo, indique por favor qué tipo de actividades realiza (puede marcar varias opciones)

- Trotar o correr
- Correr a máxima velocidad (esprintar)
- Ejercicio de fuerza (saltos, flexiones, etc.)
- Estiramientos y movilidad articular
- Ejercicios de coordinación y equilibrio
- Técnicas de manejo de la respiración (activación/relajación, conciencia)
- Otras

En caso positivo, ¿podría indicar el motivo o los motivos para reactivarse físicamente durante el descanso entre mitades?

*Tu respuesta*

En caso negativo, ¿podría indicar el motivo o los motivos para no reactivarse físicamente durante el descanso entre mitades?

*Tu respuesta*

En relación a los ejercicios de estiramiento y/o movilidad articular, ¿cuándo los realiza? (puede señalar varias opciones) *

- Nunca
- Durante el calentamiento inicial
- En el descanso entre primer y segundo cuarto
- Durante el descanso entre mitades
- En el descanso entre tercer y último cuarto

Al finalizar el Partido

Finalmente, si ha contestado que nunca realiza ejercicios de estiramiento y/o movilidad articular, ¿podría indicar el motivo/s de esto?

*Tu respuesta*

**Agradecimientos**

Como ya comentamos anteriormente, agradecemos la difusión de este formulario entre los árbitros que conozca para poder llegar al mayor número posible de personas del colectivo.

Os facilitamos el siguiente apartado para aquell@s que queráis hacer alguna aportación al proyecto podáis comentar dudas o sugerencias.

¡Muchas gracias!

Contacto: [dgonzd02@estudiantes.unileon.es](mailto:dgonzd02@estudiantes.unileon.es)

**Comentarios**

*Tu respuesta*
